# Software and Hardware Complex for Assessment of Cerebral Autoregulation in Real Time

**DOI:** 10.3390/s25196060

**Published:** 2025-10-02

**Authors:** Vladimir Semenyutin, Valeriy Antonov, Galina Malykhina, Anna Nikiforova, Grigory Panuntsev, Vyacheslav Salnikov, Anastasiya Vesnina

**Affiliations:** 1 Almazov National Medical Research Centre, Saint Petersburg 197341, Russia; nkfrv_a@mail.ru (A.N.); gpanun@mail.ru (G.P.); anastasya.vesnina@mail.ru (A.V.); 2 Institute of Computer Science and Cybersecurity, Graduate School of Computer Technologies and Information Systems, Peter the Great St. Petersburg Polytechnic University, Saint Petersburg 195251, Russia; antonov_vi@spbstu.ru (V.A.); zvs@mail.ru (V.S.)

**Keywords:** Dopplerography, cerebral autoregulation, hardware and software complex, wavelet analysis

## Abstract

The phase shift (PS) between spontaneous slow oscillations of cerebral and systemic hemodynamics reliably reflects the state of cerebral autoregulation (CA). However, CA measurements are performed retrospectively after studying the signals from the analysis sensors. At the same time, CA-oriented therapy is becoming increasingly important with the receipt of data on the state of CA in real time, especially in intensive care units. We offer a hardware and software complex for transcranial Dopplerography, which uses a non-invasive method and allows for continuous measurement of cerebral blood flow to assess the rate of CA in real time. The hardware and software complex uses sensors to measure the PS between spontaneous slow oscillations of blood flow velocity (BFV) in the middle cerebral arteries (MCAs) and systemic arterial pressure (BP) in the Mayer wave range and performs wavelet analysis of sensor signals. An examination of 30 volunteers, with an average age of 28 ± 8 years, and 15 patients, with an average age of 57 ± 16 years, with various neurovascular pathologies confirms the feasibility of using the developed hardware and software complex for continuous monitoring of PS in real time to study the mechanisms of cerebral blood flow regulation.

## 1. Introduction

Cerebral autoregulation (CA) is a physiological mechanism that ensures the constancy of cerebral blood flow with changes in cerebral perfusion pressure. Studying CA state using invasive methods is quite subjective and unsafe. Therefore, the use of invasive methods in clinical practice is limited [1]. The advent of transcranial Dopplerography and noninvasive CA assessment methods developed on its basis made it possible to conduct studies directly at the patient’s bedside at various stages of treatment [2,3,4,5,6]. The rapid development of digitalization in medicine and the use of computers and modern software packages make it possible to develop and apply methods of mathematical statistics, digital signal processing, and machine learning in medical research to study CA state [7,8,9,10,11,12,13].

It is known that phase shift (PS) of spontaneous slow oscillations of cerebral and systemic hemodynamics reflects the state of CA reliably. However, this assessment of CA is carried out retrospectively after the studies performed with the help of a short-time Fourier transform [10,14,15,16,17,18,19]. At the same time, CA-oriented therapy is becoming increasingly important with obtaining data about the state of CA in real time.

Due to the advent of modern methods of processing, including artificial intelligence, it is possible to increase the scope of non-invasive methods of measurement and control of regulatory processes. The implementation and use of shared and personalized heterogeneous data is a complex task, the solution of which must comply with accepted ethical and legal standards.

The possibility of using the wavelet transform of blood flow velocity (BFV) and blood pressure (BP) signals to assess coherence and PS in the wavelet decomposition spaces corresponding to the Mayer wave range is shown [20,21,22,23,24,25,26,27,28,29].

The development of this diagnostic method soon is very promising. A hardware and software complex developed by our group in 2019–2022 makes it possible to diagnose an impairment of CA based on the function of wavelet coherence and PS of BFV and BP signals in the wavelet decomposition spaces corresponding to the Mayer wave range and the implementation of this algorithm in a real time measuring information system [29].

Many publications are devoted to the use of the infrared spectroscopy method. This measurement method seems to be less informative than transcranial Doppler sonography, since it is based on recording the blood filling of areas of the scalp from the external carotid artery. In the process of processing infrared spectroscopy signals, the Pearson cross-correlation coefficient is usually used [30]. However, the Pearson correlation criterion provides less accurate localization of Mayer waves in time, resulting in gaps or false detections of coherence areas on which measurements are performed.

Signal processing methods with an assessment of the PS between the Mayer waves of the BP and BFV using short-term Fourier and wavelet transforms seem to be more informative. Moreover, the use of the wavelet transform and wavelet trees allows improving the localization of Mayer waves.

The study presented in the article is aimed at solving the fundamental problem of predicting the development of pathology using data analysis methods. Improving the quality of diagnostics and the effectiveness of patient treatment in the context of intensive development of high information technologies, primarily the method of signal processing and artificial intelligence, largely depends on the degree of adaptation of these technologies to specific practical goals and objectives of clinical medicine.

Carrying out the treatment and diagnostic process is impossible without a deep understanding of pathophysiological processes, the study of which involves assessing the indicators of cerebral and systemic hemodynamics. The objective of the project is to create a new technology for assessing CA in real time using measurement methods, digital signal processing, and artificial intelligence to assess the risks of cerebrovascular accident in neurosurgical patients based on an interdisciplinary approach involving specialists in the fields of measurement theory, signal processing, higher mathematics, pathophysiologists in the field of studying the pathology of cerebral circulation, and clinicians in the field of neurosurgery.

## 2. Materials and Methods

### 2.1. Test Data for CA Evaluation

Thirty volunteers aged 24 to 58 years from students and staff of the Almazov National Medical Research Center, Saint Petersburg, Russia, who did not have any cardiovascular, pulmonary, or cerebrovascular pathology, and 15 patients aged 41 to 61 years with various neurovascular pathology, including atherosclerotic carotid artery stenosis and occlusion and cerebral arteriovenous malformations, were studied. We performed non-invasive monitoring of BP using digital photoplethysmography (CN Systems Medical Technology GmbH, Graz, Austria) and BFV in both middle cerebral arteries (MCAs) using transcranial Doppler ultrasonography (MultiDop X, DWL, Sipplingen, Germany) in the supine position under the control of end-tidal CO_2_.

To assess the impact of changes in the CA state, we used hypercapnic and hypocapnic tests. The hypercapnic test consists of breathing a 5% mixture of CO_2_ with air for two minutes. The hypocapnic test includes hyperventilation of the lungs with rapid deep breathing for 1–2 min, which leads to a significant decrease in the CO_2_ content in the exhaled air. Therefore, the hypocapnic test provides a reliable decrease in BFV and an increase in the tone of the distal arteries and arterioles.

The patient’s screening protocol was approved by the Ethics Board of the Almazov National Medical Research Center (Protocol No. 1 dated 02.06.2010). All patients who took part in the clinical study signed a written voluntary informed consent. The study complied with the Declaration of Helsinki adopted by the World Medical Association. No animal studies or human experiments were conducted as part of this work.

### 2.2. Time-Frequency Analysis of CA Characteristics

The numerical method for assessing the state of the CA is based on determining the time intervals of consistency between the BP and BFV fluctuations in the Mayer waves range. Physiological processes of autoregulation do not manifest themselves constantly, but they are observed at limited time intervals in the form of coordinated fluctuations in BP and BFV at a certain frequency $f_{M}$ belonging to the range of Mayer waves $f_{M}$ ∈ [50 mHz–150 mHz]. The task of analyzing the target audience is reduced to the digital processing of the BP and BFV signals to determine the following characteristics:

- time intervals at which consistent oscillations of the BP and BFV occur in the range of Mayer waves;
- a specific frequency $f_{M}$ of the coordinated oscillations of the BP and BFV from the range of Mayer waves;
- coherence function of the BP and BFV oscillations at the frequency $f_{M}$;
- PS between BP and BFV at the frequency $f_{M}$.

The solution to these problems is based on the time-frequency analysis of the BP and BFV signals in real time with high resolution in both time and frequency domains. The methods of short-time Fourier transform and wavelet transform of signals have a high resolution in the time and frequency domains and therefore meet the task.

#### 2.2.1. Short-Time Fourier Transforms

Locally stationary signals BP and BFV have frequency characteristics that change over time. During the observation time, oscillations in the Mayer range can arise and cease, the oscillation frequency $f_{M}$ can change within the Mayer range, and the coherence property and PS between oscillations also change. The Short-Term Fourier Transform (STFT) can be used to evaluate locally stationary signals [18].

The STFT transformation is performed on short time intervals within a frame. The frame is shifted in time by selecting signal samples in accordance with the frame length $N_{frame}$, which are denoted as *x* $x\left(n\right),y\left(n\right),n=1,\ldots ,N_{frame}$. The signals x(n) and y(n) are a mixture of useful signals, BP and BFV characterizing the CA, with other physiological signals and random noise ζ(n). To suppress noise, each data frame is divided into L windows of length $N_{win}$. The window size is several times smaller than the frame size, which allows averaging the results within the frame. STFT is calculated in each rectangular window based on signal samples $x\left(n\right),y\left(n\right),n=1,\ldots ,N_{win}$. The rectangular window can be shifted by half its length $N_{shift}=\frac{1}{2}N_{win}$ without introducing additional losses.

Within each window, the signals are centered, and the discrete Fourier transform is calculated over the number of samples $N_{win}$, the result of which is the frequency characteristics $X\left(k\right),Y(k)$ depending on the discrete frequencies k. The mutual spectral density of the signals, Sx,yk=X*kY(k)Nwin, the modulus of the spectral density $\left|S_{x,y}\left(k\right)\right|$, and the phase between the signals $\theta_{x,y}$ are determined according to the equation:

(1) $$S_{x,y}\left(k\right)=\left|S_{x,y}\left(k\right)\right|\exp\left(-j\theta_{x,y}\right).$$

We determine the proper spectral densities of signals *x*(*n*), *y*(*n*) within each window:

(2) $$S_{x,x}\left(k\right)=\frac{X(k)X^{*}(k)}{N_{win}};$$

(3) $$S_{y,y}\left(k\right)=\frac{Y(k)Y^{*}(k)}{N_{win}};$$

The calculations are repeated when the window is shifted by half its length. As a result of averaging over windows, the spectral densities of signals within a frame contain reduced noise. The averaging of spectral densities for each frame is calculated using the following formulas:

(4) S^x,xk=1L∑i=1LSx,xik;   S^y,yk=1L∑i=1LSy,yik;S^x,yk=1L∑i=1LSx,yik.

where ${\hat{S}}_{x,x}\left(k\right),\;{\hat{S}}_{y,y}\left(k\right)$ are the average values of the spectral density of signals *x*(*n*) and *y*(*n*) for the current frame, ${\hat{S}}_{x,y}\left(k\right)$ is the average value of the joint spectral density of signals *x*(*n*) and *y*(*n*).

Using the average spectral densities (4), we calculate the coherence function between signals for each frame using the formula:

(5) $${\hat{\theta }}_{x,y}\left(k\right)=\sqrt{\frac{{\left|{\hat{S}}_{x,y}\left(k\right)\right|}^{2}}{{\hat{S}}_{x,x}\left(k\right){\hat{S}}_{y,y}\left(k\right)}}.$$

Let us determine $k_{M}$—the discrete Mayer frequency at which the coherence value between the BP and BFV signals reaches its maximum value:

(6) $$k_{M}=argmax\left({\hat{\theta }}_{x,y}\left(k\right)\right).$$

The average value of PS ${\hat{\gamma }}_{x,y}(k_{M})$ between the BP and BFV signals for a discrete frequency $k_{M}$ can be determined by the following relationship:

(7) $${\hat{S}}_{x,y}\left(k_{M}\right)=\left|{\hat{S}}_{x,y}\left(k_{M}\right)\right|\exp\left(-j{\hat{\theta }}_{x,y}(k_{M})\right)$$

For better noise suppression, the coherence function ${\hat{\gamma }}_{x,y}\left(k_{M}\right)$ and the PS ${\hat{\theta }}_{x,y}(k_{M})$ are calculated for each subsequent frame shifted relative to the previous frame by the window size. Thus, each frame is updated with data from one subsequent window. For each current *m*-th frame, we obtain the coherence function ${\hat{\gamma }}_{x,y}\left(k,m\right)$, and the PS function ${\hat{\theta }}_{x,y}(k_{m},m)$, where $k_{m}$ is the frequency sample number and m is the time sample number. The implemented method allows obtaining a time resolution equal to half the window Nshift=Nwin2. Thus, the result of the algorithm is a two-dimensional frequency-time function of signal coherence $\gamma_{x,y}\left(k_{M},m\right)$ and a two-dimensional time-frequency function of phase shift ${\hat{\theta }}_{x,y}(k,m)$ at the Mayer frequency. If the value of the square of coherence exceeded the threshold value ${\hat{\gamma }}_{x,y}^{2}(k_{M},m)\geq th$, then we proceeded to the calculation of PS. The threshold th = 0.6 was chosen empirically by analyzing data for healthy volunteers and patients. Experience has shown that it is acceptable to vary the threshold value in the range of 0.6–0.75. The threshold value of 0.6 is consistent with the recommendations of other researchers. However, the program allows you to change the threshold.

**Figure 1:**
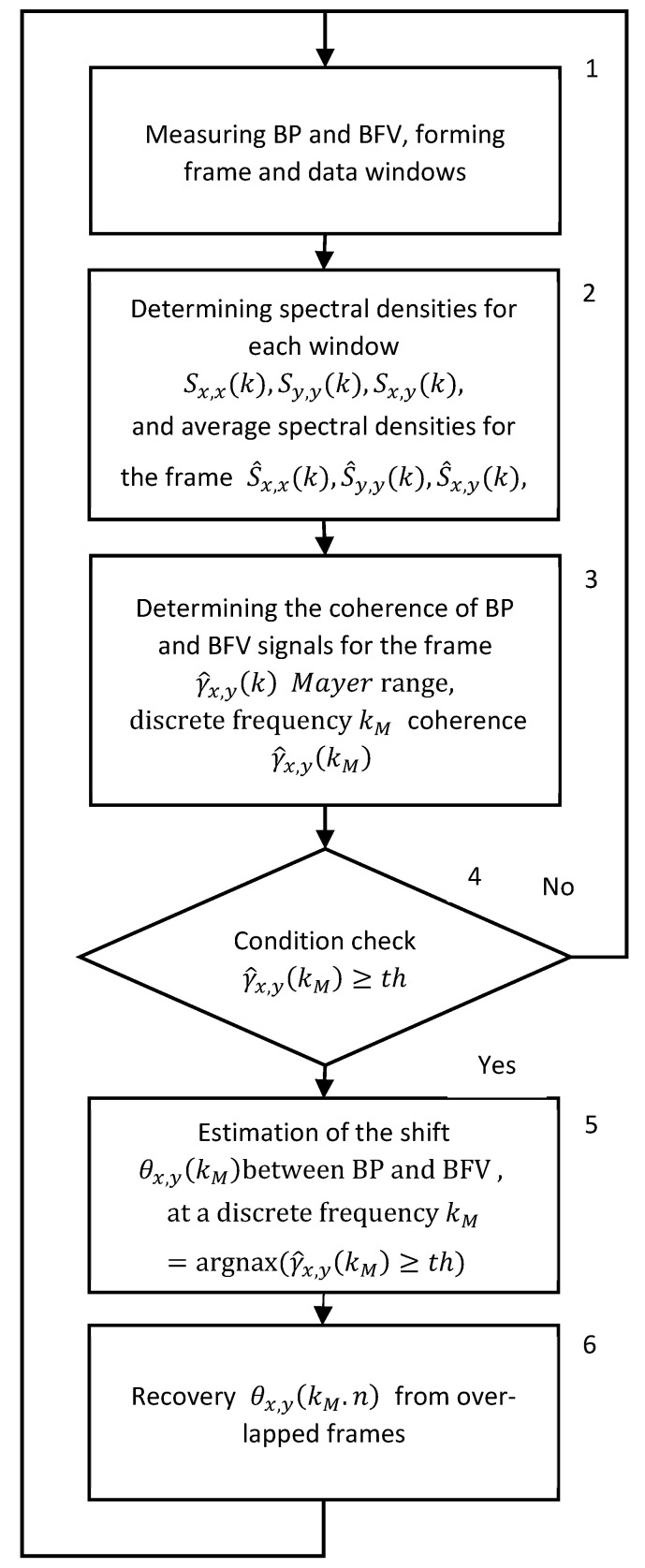
Generalized algorithm for real time STFT analysis of signals from photo and ultrasonic sensors of BP and BFV.

A generalized algorithm for real time BP and BFV analysis using STFT is presented in Figure 1. The measurement of BP and BFV is performed synchronously with an interval of 0.01 s. The measurements form frames and windows (block 1). In block 2, the STFT is calculated for each window, and their average values are determined within the frame. In block 3, the coherence function, the phase shift function, the averaged spectrum in the Mayer wave range, and the coherence and phase shift values are calculated. In blocks 4 and 5, the PS is calculated if the square of the coherence exceeds the threshold value. In block 6, the current value of the phase shift is restored from the overlapping windows.

The frame size for the STFT was $N_{frame}=16,384$ samples with sample rate Δt=0.01 s, and the frame duration $T_{frame}=163.84\;s$. Inside the frame, the sliding Hamming window was used, which size is $N_{win}=2048$ samples $T_{win}=20.48\;s$, with offset 512 samples (5.12 s). Accordingly, the number of windows inside the frame is 32.

When analyzing the state of the CA in real time, an important characteristic is the resolution in time and frequency. Time resolution is determined by the duration of the frame $T_{frame}=163.84\;s$, the value of which affects the definition of coherence intervals. The frequency resolution of 0.048 Hz, which depends on the duration of the window $T_{win}=20.48\;s$, is important for fine-tuning the Mayer frequency, at which the coherence of the BP and BFV signals is determined. The use of the STFT with averaging the results of the windows within the frame makes it possible to obtain a constant resolution in time and frequency over the entire range of signal frequencies, which does not always provide the best results of time-frequency analysis. Thus, when using the STFT, the need to suppress interference present in the physiological signals of BP and BFV leads to a decrease in the resolution in time and frequency [22].

#### 2.2.2. Continuous Wavelet Transform (CWT)

An important problem with STFT is the choice of window length. A relatively large window allows for more accurate determination of the Mayer frequency and phase shift. A small window allows for more accurate determination of the boundaries of coherence intervals, but short intervals may be missed [19]. Thus, the window size determines the balance between accuracy and locality. The CWT method eliminates this drawback of STFT. Therefore, it is advisable to use CWT with a harmonic wavelet function to detect coherence and phase shift of the harmonic components of BP and BFV signals [20]. The group of harmonic wavelets includes the complex Morlet wavelet, the complex Paula wavelet, and the difference of Gaussians wavelet.

The CWT of the signal $x\left(n\right),\;n=t/\delta t$ sampled with an interval of δt is determined as a convolution with a scaled and normalized wavelet $\frac{(n-n^{\prime })\delta t}{s}$:

(8) $$X\left(s,n\right)=\sum_{n^{\prime }=0}^{N-1}x(n)\psi^{*}\frac{(n-n^{\prime })\delta t}{s},$$

where $(n-n^{\prime })\delta t$ is the parameter corresponding to the time shift, s is the parameter that determines the scaling, and $\psi^{*}(.)$ is the complex conjugation.

A more efficient way to calculate wavelet coefficients is to use the convolution theorem. The calculation involves the inverse Discrete Fourier Transform (DFT) of the product of the DFT of the signal x(n) and the wavelet ψ(n,s) using the formula:

(9) $$X\left(s,n\right)=x\left(n\right)\cdot \psi \left(n\right)=F^{-1}\left[F\left(x\left(n\right)\right)\cdot F\left(\psi \left(n,s\right)\right)\right],$$

where $F^{-1}$ is the inverse DFT operator.

We chose the Morlet wavelet with parameter $\omega_{0}=6$ which are often used in research:

(10) $$\psi \left(t\right)=\pi^{-\frac{1}{4}}e^{-i\omega_{0}t}e^{-\frac{t^{2}}{2}}.$$

This wavelet allows for rapid determination of changes in the spectrum of a non-stationary signal and is therefore most often used in medicine to analyze cardiograms and encephalograms. In the frequency domain, the Morlet wavelet has the following form:

(11) $$\hat{\psi }\left(s\omega \right)=\pi^{-\frac{1}{4}}e^{-\frac{{\left(s\omega -\omega_{0}\right)}^{2}}{2}}.$$

### 2.3. Wavelet Transform of Signals Characterizing CA

The characteristics of the CA are the PS between BP and BFV, obtained in the space of the wavelet decomposition on scales corresponding to the range of Mayer waves, under the condition of coherence of these signals.

Sequences of BP and BFV signals obtained from photo- and ultrasonic sensors at discrete time $x^{\prime }(n)\;и\;y^{\prime }(n)$, form frames. Within the frames, the signals are centered $x(n)=x^{\prime }\left(n\right)-\frac{1}{N}\sum_{n=0}^{N-1}x^{\prime }\left(n\right)$, ${y\left(n\right)=y}^{\prime }\left(n\right)-\frac{1}{N}\sum_{n=0}^{N-1}y^{\prime }\left(n\right)$. The frame duration V is determined by the formula: $V=2^{floor\left({log}_{2}(N^{\prime })\right)+1}$, where the initial frame length $N^{\prime }$ increases to the nearest power of two, exceeding $N^{\prime }$.

For each frame of BP and BFV, we perform DFT:

(12) $$X(k)=\sum_{n=0}^{N-1}x(n)e^{-i2\pi kn},$$

(13) $$Y(k)=\sum_{n=0}^{N-1}y(n)e^{-i2\pi kn},$$

where k=0, 1, 2,…,N−1 frequency index, *X*(*k*)—Fourier image of the BP signal, *Y*(*k*)—Fourier image of the BFV signal in the frame.

The wavelet decomposition represents signals in the frequency-scale space. The minimum scale is determined by the sampling interval $s_{0}=2\delta t;$ the current decomposition levels j = 0, 1, 2,…,J correspond to the scales $s_{j}=s_{0}2^{j\delta_{i}},\;J=\delta j^{-1}{log}_{2}\left(\frac{N_{x}\delta t}{s_{0}}\right).$ Each decomposition scale $s_{j}$ is associated with a pseudo-frequency $f_{i}=\frac{\omega_{0}}{2\pi s_{i}}$ using the Fourier coefficient $\eta_{0}=\frac{\omega_{0}}{2\pi }.$ Then the circular frequency $\omega_{k}$ at each decomposition scale is determined by the formula:

(14) $$\omega_{k}=\left\{\begin{array}{cc} \frac{2\pi k}{N\delta t}, & k\leq \frac{N}{2}\\ -\frac{2\pi k}{N\delta t}, & k>N \end{array}\right.$$

The parameter $\omega_{0}$ of wavelet determines the resolution of the wavelet transformation in time and frequency. For the CA analysis, we chose the Morlet wavelet with the parameter $\omega_{0}=6$, which approximately corresponds to the respiratory rate.

The analytical Morlet wavelet $\hat{\psi }\left(s\omega \right)=\pi^{-\frac{1}{4}}exp\left(-\frac{{\left(s\omega -\omega_{0}\right)}^{2}}{2}\right)H\left(\omega \right),$ includes the Heaviside function, which is defined for the frequency $\omega_{k}$ $H\left(\omega_{k}\right)=\left\{\begin{array}{cc} 1, & \omega_{k}>0\\ 0, & \omega_{k}\leq 0 \end{array}\right.$. At each scale of the decomposition, the wavelet coefficients normalize $\hat{\psi }\left(s\omega_{k}\right)={\left(\frac{2\pi s}{\delta t}\right)}^{\frac{1}{2}}{\hat{\psi }}_{0}\left(s\omega_{k}\right).$

The coefficients of the wavelet decomposition of discrete signals x(n),y(n) are determined in the frequency domain by means of element-wise multiplication of the Fourier transformation of the analyzed signal X(k) и Y(k) by the Fourier transforms of the wavelets ${\hat{\psi }}^{*}\left(s\omega_{k}\right)$ at each level of decomposition. The subsequent inverse DFT transform allows us to obtain the wavelet coefficients:

The coefficients of the wavelet decomposition of discrete signals $x\left(n\right),\;y(n)$ are determined in the frequency domain by element-wise multiplication of the Fourier transform of these signals X(k) and Y(k) by the Fourier transform of the wavelets ${\hat{\psi }}^{*}\left(s\omega_{k}\right)$ at each level of decomposition. The subsequent inverse Fourier transform allows us to obtain the wavelet coefficients using the formula:

(15) $$c_{x}\left(n,s\right)=\sum_{n=0}^{N-1}X(k){\hat{\psi }}^{*}\left(s\omega_{k}\right)e^{i\omega_{k}n\delta t}$$

(16) $$c_{y}\left(n,s\right)=\sum_{n=0}^{N-1}Y(k){\hat{\psi }}^{*}\left(s\omega_{k}\right)e^{i\omega_{k}n\delta t}$$

where $c_{x}\left(n,s\right),\;c_{y}\left(n,s\right)$ are the wavelet coefficients obtained for discrete time n and scale s.

Calculation of the wavelet coefficients of the Fourier domain makes it possible to reduce the complexity of calculations. To reduce noise, we smooth the coefficients $c_{x}\left(n,s\right)$ and $c_{y}\left(n,s\right)$ in the time and scale domain. Time smoothing is performed with a Gaussian filter on each scale $s=1:N_{s}.$ Scale smoothing is performed by an averaging filter. Therefore, we obtain the wavelet coefficients of two signals $x\left(n\right)\;and\;y(n)$ smoothed in time and scale domains.

The wavelet cross spectrum characterizes the total mutual energy of two signals, which is nonzero if the two signals are correlated with each other, and allows us to show when and at what frequencies the signals are synchronized:

(17) $$c_{x,y}\left(n,s\right)=c_{x}^{*}\left(n,s\right)c_{y}\left(n,s\right).$$

The cross spectrum of a wavelet is presented as a product of the amplitude and phase spectra:

(18) $$c_{x.y}\left(n,s\right)=\left|c_{x.y}\left(n,s\right)\right|\exp\left(arctg\frac{Im\left(c_{x,y}\left(n,s\right)\right)}{Re\left(c_{x,y}\left(n,s\right)\right)}\right).$$

The modulus of the normalized cross spectrum characterizes the coherence or consistency of two signals. The normalized coherence value changes from zero to one and determines a linear relationship between the BP and BFV. The square of the normalized coherence value is determined by the formula:

(19) $$H_{x,y}^{2}\left(n,s\right)=\frac{{\left|c_{x}^{*}\left(n,s\right)c_{y}\left(n,s\right)\right|}^{2}}{{\left|c_{x}^{*}\left(n,s\right)\right|}^{2}{\left|c_{y}\left(n,s\right)\right|}^{2}}.$$

Coherence is defined in the scale space corresponding to $s_{M}$—the Mayer pseudofrequency. The presence of coherence of the BP and BFV signals is determined by two conditions: coherence in the $s_{M}$ space is maximum in the range of Mayer pseudofrequencies and exceeds the threshold value $H_{x,y}^{2}\left(n,s_{M}\right)\geq th.$ The threshold value was chosen empirically based on data from tests of healthy volunteers and patients. We varied the threshold from 0.5 to 0.75 and recorded the coherence intervals. The best results were obtained for the threshold of th=0.6.

The local phase shift of the signals can be obtained from the following formula:

(20) $$\theta_{x,y}\left(n,s_{M}\right)=arctg\frac{Im\left(c_{x,y}^{*}\left(n,s_{M}\right)\right)}{Re\left(c_{x,y}^{*}\left(n,s_{M}\right)\right)}.$$

The threshold coefficient for the coherence value equal to 0.6 corresponds to the decorrelation length for the Morlet wavelet function in the $s_{M}$ space [21,22,23].

**Figure 2:**
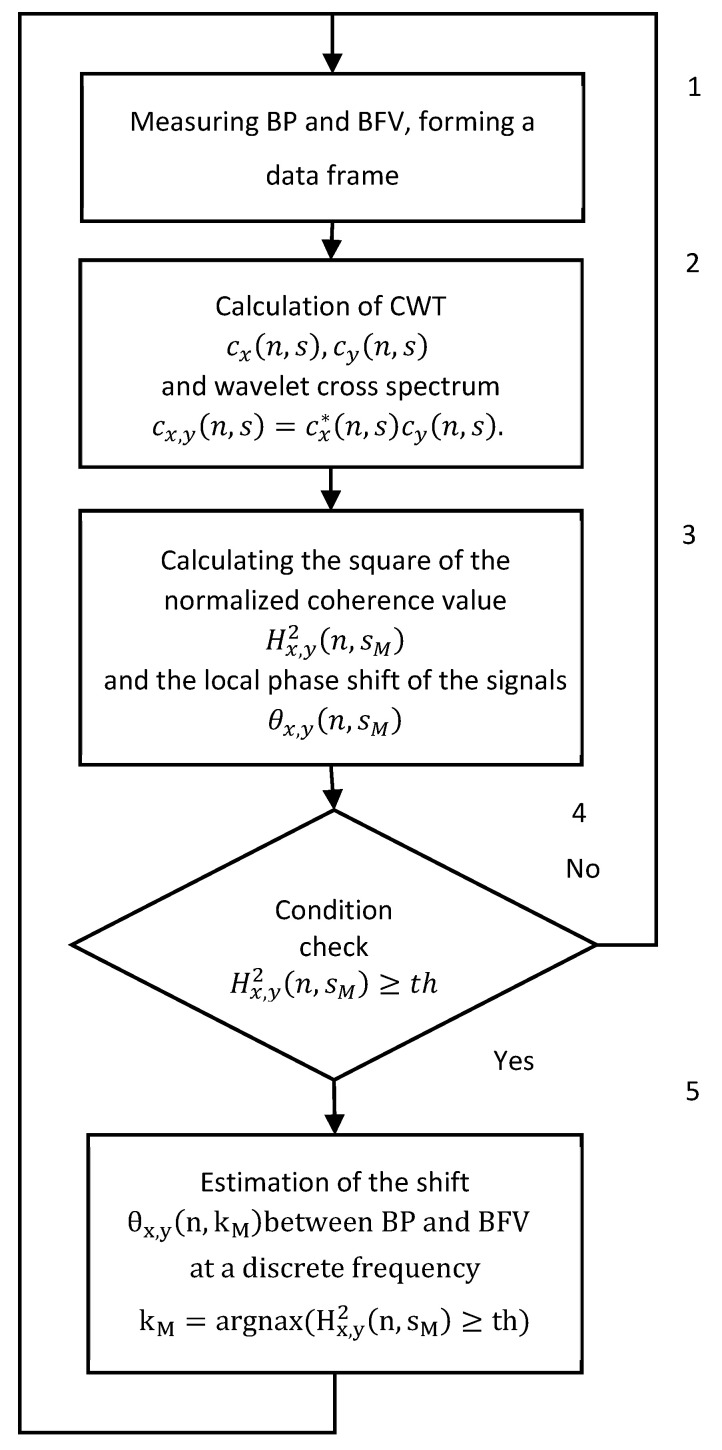
Generalized algorithm for real time CWT analysis of signals from photo and ultrasonic sensors of BP and BFV.

The measurement of the BP and BFV is performed synchronously with an interval of 0.01 s. A generalized algorithm for real-time CWT analysis is shown in Figure 2. Block 1 receives the measurement results and forms frames. Block 2 performs the digital wavelet transform and calculates the cross-spectrum of wavelets for each frame of the BP and BFV data. Block 3 calculates the coherence function and the phase shift function of the wavelets. Block 4 checks the condition of the squared coherence exceeding the threshold value. If the threshold is exceeded, the algorithm calculates the PS; otherwise, the PS is not calculated, and the calculations proceed to the next frame (block 5).

Signal processing and calculations were performed on the Matlab-2019R platform. The significance of differences in values was assessed using the Student’s *t*-test. The sensitivity of the PS to changes in the state of the spacecraft is assessed by the relative phase shift: *η* = : η= θext−θ¯θ¯ , where $\theta_{ext}$ is the interval of absence of autoregulation of $\bar{\theta }$. The stand-alone application was written in the C programming language to obtain an acceptable calculation result in real time. To obtain a more user-friendly interface, we used the Windows/CVI software development environment from National Instruments Lab (2024). For smaller samples of examined individuals, the reliability of differences between the methods based on CWT and STFT was determined using the Student’s *t*-test.

## 3. Results

**Figure 3:**
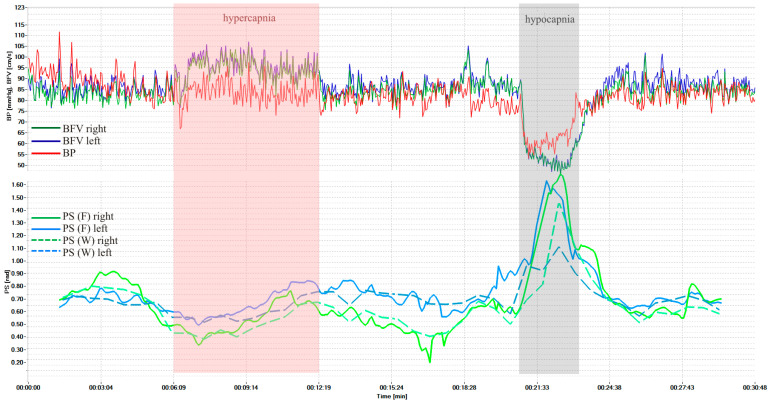
BP and BFV in the MCA and PS obtained using STFT and CWT under standardized loads (hypercapnic and hypocapnic tests of a healthy volunteer, variant 1).

**Figure 4:**
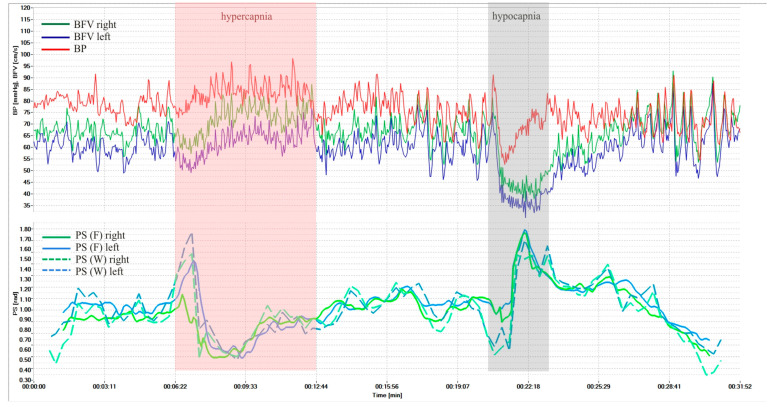
BP and BFV in the MCA and PS obtained using STFT and CWT under standardized loads (hypercapnic and hypocapnic tests of a healthy volunteer, variant 2).

Figure 3 shows dynamics of BP and BFV in both MCA and PS by Fourier and wavelet transform during standardized load—hypercapnic and hypocapnic tests in healthy volunteers. These loads led to a significant decrease and increase of PS, and it is a normal reaction for all volunteers. Figure 4 shows another example of the results of a healthy volunteer, but with more pronounced changes in the wavelet transformation.

The average PS values using Fourier transform in healthy volunteers were 0.98 ± 0.28 rad on the left and 0.99 ± 0.26 rad on the right. Against the background of hypercapnia, the average minimum value of PS was 0.46 ± 0.23 rad (*p* = 3.8 × 10^−11^) on the left and 0.45 ± 0.22 rad (*p* = 3.9 × 10^−12^) on the right; against the background of hypocapnia, the average maximum value of PS was 1.37 ± 0.31 rad (*p* = 2.9 × 10^−10^) on the left and 1.37 ± 0.31 rad (*p* = 4.8 × 10^−10^) on the right.

The average PS values using the wavelet transform in healthy volunteers were 0.91 ± 0.26 rad on the left and 0.92 ± 0.28 rad on the right. Against the background of hypercapnia, the average minimum value of PS was 0.44 ± 0.22 rad (*p* = 3.6 × 10^−11^) on the left and 0.42 ± 0.23 rad (*p* = 2.9 × 10^−11^) on the right; against the background of hypocapnia, the average maximum value of PS was 1.25 ± 0.26 rad (*p* = 2.7 × 10^−14^) on the left and 1.30 ± 0.28 rad (*p* = 1.8 × 10^−13^) on the right.

The comparison results of the proposed method using a cross-wavelet spectrum with the method based on the short-time Fourier transform showed that the proposed method has a higher sensitivity to changes in the rate of CA and better localization of changes in time and frequency.

**Figure 5:**
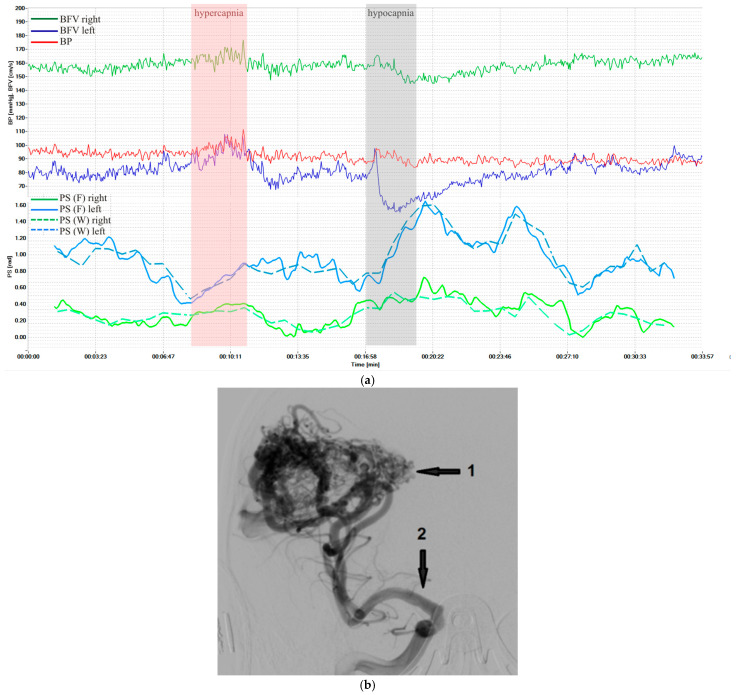
Characteristics of a patient with cerebral arteriovenous malformation supplied from the right middle cerebral artery (MCA): (**a**) BP and BFV in the MCA and PS obtained using STFT and CWT under standardized loads; (**b**) carotid angiography: the arrows indicate AVM nidus (1) and afferent vessel—right MCA (2).

Figure 5 shows results in a patient with cerebral arteriovenous malformation during similar loads. However, perverse reactions to hypercapnia were observed, probably associated with the absence of reaction of pathological vessels to CO_2_.

All examined patients with various neurovascular pathology, atherosclerotic carotid artery stenosis and occlusion, and cerebral arteriovenous malformations had asymmetry in CA indicators and different dynamics of PS during standardized loads.

STFT and CWT have the same data frame duration of 163.84 s. Several factors influence the results of determining the state of the CA using the proposed algorithms:

- correctness of classification of intervals of presence or absence of coherence of BP and BFV signals;
- accuracy of determining the coherence of signals in the frequency range of interest;
- estimation accuracy of the Mayer waves range corresponding to the maximum coherence in the interval (50 mHz–150 mHz);
- estimation accuracy of the PS between the BP and BFV signals at the Mayer waves range.

In turn, the above indicators of the accuracy of the algorithms for determining the state of the CA depend on the achieved resolution in time and frequency.

To suppress noise, the STFT algorithm uses averaging of results within a frame using 32 consecutive windows with a duration of 20.48 s. The time resolution is determined by the frame duration $T_{frame}=163.84\;s$. The frequency resolution depends on the window duration, at $T_{win}=20.48\;s$ it is 0.048 Hz. Averaging of STFT results within a frame allows us to suppress noise. The time and frequency resolution are slightly degraded and remain constant over the entire frequency range of the signal.

After performing CWT, we use smoothing filters to suppress noise in both the time domain and the pseudo-frequency domain. The presence of filters affects the time and frequency resolution. CWT uses 12 votes, which allows us to determine 14 pseudo-frequencies in the Mayer wave band. The length of the smoothing filter in the pseudo-frequency domain is 12 samples. The criterion for choosing a pseudo-frequency subband corresponding to the Mayer wave is the maximum coherence of wavelets. In this subband, the largest proportion of measurements is suitable for calculating the PS. When using the CWT algorithm, the frequency resolution depends on the time resolution. On average, in the Mater waves range, the frequency resolution is 0.006 Hz. When scanning the Mayer wave subbands, the relative duration of the coherence intervals was in the range from 0.35 to 0.88.

Thus, the higher frequency and time resolution of the CWT algorithm improves the results of the CA study.

## 4. Discussion

Currently, non-invasive assessment methods based on retrospective cross-spectral and cross-correlation analysis of slow oscillations of systemic and cerebral hemodynamics are used to assess CA in humans under normal conditions and in various pathological conditions. When diagnosing emergency conditions in intensive care settings, it is necessary to use a method that would provide operational information about the CA state in real time, with better time and frequency resolution.

In our previous works [24,25], we proposed an algorithm based on short-term cross-Fourier spectrum and coherence spectrum, which can obtain the estimated characteristics with a constant scale resolution in time and frequency. The results obtained with standardized loads on the cerebral circulatory system showed the possibility of assessing the CA state in real time and for the first time established the advantages of wavelet analysis for collecting reliable data on the PS between the Mayer waves of BFV and BP, allowing monitoring both with a higher sensitivity and with better resolution in the time-frequency domain due to the use of continuous wavelet transform of signals.

However, at certain time intervals the condition may not be met, there is no coherence, and the phase shift cannot be calculated. In such cases, the graphs (Figure 3, Figure 4 and Figure 5) may have gaps, the number of which should be reduced. To do this, we propose to use a more accurate approximation of the Mayer frequency. In our case, the wavelet transforms using 12 voices allow us to search at 14 pseudo-frequencies in the Mayer waveband. The criterion for selecting a subrange may be the largest proportion of values suitable for calculating the phase shift. When scanning subranges of Mayer waves, it turned out that the relative proportion of coherence intervals varies from 0.35 to 0.82 for BP and BFV in the left hemisphere and from 0.50 to 0.88 for BP and BFV in the right hemisphere. Thus, increasing the frequency resolution improved the results of the study.

Examinations of healthy volunteers and patients with CA disorders, carried out using the hardware and software complex, showed the reliability of the results of non-invasive assessment of CA velocity in real time. The developed analytical measuring system, through simultaneous monitoring of systemic and cerebral hemodynamics and CA velocity, made it possible in real time to increase non-invasiveness, the effectiveness of an objective assessment of a person’s normal condition, and the identification of a group of patients with CA disorders and a high risk of complications. All this can determine the tactics and constant monitoring of treatment results. The average maximum values of PS during standardized hyper- and hypocapnic loads in healthy volunteers and patients with various cerebral vascular pathologies correspond to literature data [2,10,12,13]. At the same time, the dynamics of PS before, during, and after the tests were different (Figure 3 and Figure 4) and, perhaps, characterize the state of CA at each time point and require further study to clarify the mechanisms of CA in normal conditions and in diseases of the cerebral vessels.

The accumulation and centralization of digitized research data from many patients, as well as the consolidation and formation of a knowledge base in the field of medicine and physiology, can serve as a prerequisite for further research. Observations of one patient contribute to a deeper understanding of disease mechanisms and hidden patterns, where machine learning techniques can be used.

The authors [30] use a method for assessing CA using the correlation coefficient (Pearson), and it is devoted to the possibility of replacing the TCD (Mx) method with the uninformative method of infrared spectroscopy (COx), since it records changes in blood filling of scalp regions (the external carotid artery area). There are many articles using Mx or PRx (ICM+ system) for continuous assessment of CA, but we use a fundamentally different processing method with an assessment of the PS between the Mayer waves of BP and BFV using Fourier and wavelet transformers.

The presented article supplements the results obtained after the publication of the first article [29]. Since 2019, the Laboratory of brain circulation pathology of the Russian Polenov Neurosurgical Institute of Almazov National Medical Research Centre, together with Peter the Great St. Petersburg Polytechnic University, has been developing a hardware and software complex for recording the CA state in real time. A total of 30 volunteers and 15 patients were examined. Our first article presented the results of examining 9 patients and 3 volunteers. With a larger examination, we obtained more reliable results. For example, with hyperventilation in volunteers, the reliability of differences increased significantly from *p* = 0.014–0.022 to *p* = 0.0005–0.0008. Both articles are devoted to a completely new method of data processing. The methodology is described in detail in both articles.

BP and BFV signals measured by photo and ultrasound sensors are multicomponent, since they reflect several physiological processes. The signal components include heart rate waves, Mayer waves, B-waves, and broadband noise. Other modern adaptive methods and algorithms can be used to process multicomponent signals. In particular, the empirical mode decomposition (EMD) method is a fully data-driven adaptive process that has been successfully applied to multicomponent AM-FM signals [31]. EMD is implemented using computationally complex spline approximation methods, adaptive neural networks, pretrained neural networks, and other computationally complex methods. Blind methods of signal separation and extraction, which allow separating signals and extracting the signal of interest from a mixture [32], use an adaptive neural network. Therefore, in addition to the STFT and CWT-based methods discussed in the paper, our research group investigated the application of fractal methods for signal analysis based on the calculation of the Hölder multifractal spectrum and the correlation dimension of signals [33]. The operation of the real time measurement system requires high computational performance. The methods listed here perform complex calculations and require high computing performance. The use of the CWT with short wavelet filters significantly reduces the computational load and allows the device to be used directly at the patient’s bedside.

## 5. Conclusions

The results of the conducted studies confirm the possibility of using the software and hardware complex for continuous monitoring of PS in real time for the purpose of diagnosis and correction of treatment, including intensive care.

The proposed complex makes it possible in real time to identify a group of patients with CA impairment and a high risk of complications and to determine the tactics and prognosis of treatment results.

Certain advantages of the wavelet transform have been established when carrying out standardized loads, which makes it possible to recommend the use of the wavelet transform for constructing algorithms for processing slow oscillations of systemic and cerebral hemodynamics section may be divided by subheadings. It should provide a concise and precise description of the experimental results and their interpretation, as well as the experimental conclusions that can be drawn.

## 6. Patents

Method for assessing the state of cerebral autoregulation in real time Patent holder: Federal State Budgetary Institution “National Medical Research Center named after V.A. Almazov, ” Ministry of Health of the Russian Federation (RU) Authors: Semenyutin V.B. (RU), Nikiforova A.A. (RU), Antonov V.I. (RU), Malykhina G.F. (RU), Salnikov V.Y. (RU).

## Data Availability

The initial research data are presented in Figure 3, Figure 4 and Figure 5. The remaining research data on patients and volunteers are stored in the Archive of the Laboratory of Cerebral Circulation Pathology of the A.L. Polenov Russian Scientific Research Institute.
